# The Implications of Filial Piety in Study Engagement and Study Satisfaction: A Polish-Vietnamese Comparison

**DOI:** 10.3389/fpsyg.2020.525034

**Published:** 2021-01-26

**Authors:** Joanna Różycka-Tran, Paweł Jurek, Thi Khanh Ha Truong, Michał Olech

**Affiliations:** ^1^Institute of Psychology, University of Gdańsk, Gdańsk, Poland; ^2^VNU University of Social Sciences and Humanities, Vietnam National University, Hanoi, Vietnam; ^3^Department of Psychology, Medical University of Gdańsk, Gdańsk, Poland

**Keywords:** filial piety, DFPS, study engagement, study satisfaction, cross-culture psychology

## Abstract

Even in psychological literature, which describes many determining variables related to the school domain, few studies have investigated the universal (i.e., etic) mechanism underlying parent–child relations, which is a prototype matrix for future student–teacher relations. The role of the imprinted schema of children’s obligations toward parents seems to be crucial for school functioning in classroom society. The Dual Filial Piety Model (DFPM; Yeh, 2003) is comprised of two higher-order factors that correspond to the two focal filial piety attributes: reciprocal (need of interpersonal relatedness) and authoritarian (need of social belonging and national identity), which have been shown to have distinct implications on social adaptation and individuals’ psychological functioning. In this study, we investigate the relationship between *filial piety* and student attitudes (study engagement and satisfaction) in a more individualistic and egalitarian culture (Poland, *N* = 310) and in a more collectivistic and hierarchical society (Vietnam, *N* = 297). The measurement invariances of three scales, i.e., the Vietnamese adaptation of DFP Scale, the Utrecht Work Engagement Scale (UWES–S9), and the Study Satisfaction Scale, were improved in the MLM analyses. Our results show that in more individualistic cultures, the RFP (reciprocal mode) is a stronger predictor of study engagement and study satisfaction; however, the AFP (authoritarian mode) is a better factor to predict study engagement in more collectivistic cultures. What is more, only RFP positively correlates with study satisfaction in individualistic culture. Our findings revealed that in different cultures, different aspects of filial piety should be emphasized by parents in the context of the future academic achievements of their children. The conclusion is that the prevention and intervention strategies or techniques intended for children with school problems should be culturally appropriate and addressed to the parents of kindergarten and later to very early-stage education teachers. The results of studies based on the DFPM may stimulate practical applications and policy development within the domain of success and failure in the academic environment.

## Introduction

Nowadays, we can observe an increased interest in the topic of school achievement, as school failure seems to have become an important issue in the present world (Galindo et al., 2018). With respect to the development of prevention and intervention strategies and techniques, the factors that underline school problems and the variables responsible for academic achievement and failure, should be first investigated and diagnosed, especially in the cross-cultural context of multiculturalism and the internationalization of education.

In psychological literature describes many determining variables related with school failure and academic achievement or satisfaction, e.g.,: the influence of self-control and grit on academic self-efficacy and satisfaction with school (Oriol et al., 2017); the links between career preparedness and academic development (Oliveira et al., 2017); the relationship between emotion understanding and school achievement (Franco et al., 2017); the association between variables of individual and school-related well-being and those of school achievement and performance (López et al., 2017); and the influence of family socio-economic status and parental support on success at school (Pires et al., 2017). Although many relationships between different variables and school problems have already been established, few studies have investigated the universal (i.e., etic) psychological mechanism underlying parent–child relations, which could be applicable in any culture. The imprinted schema of a child’s obligations toward their parents seems to be a prototype for the student–teacher relationship in school society.

In this study, we investigate the relationship between *filial piety* and student attitudes (study engagement and satisfaction) in a more individualistic and egalitarian culture (Poland) and in a more collectivistic and hierarchical society (Vietnam), [see Hofstede et al. (2010) and Różycka-Tran et al. (2017)].

Filial piety is a quite modern psychological variable with origins in Asian (mainly Chinese) indigenous psychology. Filial piety was viewed as a culture-specific concept, denoting the idea of family interdependence and the close connection between children and their parents (Ho, 1994; Yeh and Bedford, 2003). Several filial piety duties have been described in the traditional culture of China, including the care and respect for and attendance upon the needs of one’s parents and the provision of physical and financial care for one’s parents (Yeh, 2003). In the classic definition, filial piety was viewed as a strong belief based on love and respect and amorally justified behavior referring to children’s attitudes about how they should treat their parents; it has thus been conceptualized in Chinese research as an indicator of parent–child interaction quality (Yeh, 2003, 2006; Wong et al., 2010; Chen and Ho, 2012).

Although the expression of affection may differ by culture, the affection-based interaction and relations between children and parents are present in all cultures. Some studies in filial attitudes and behaviors have been conducted also in non-Chinese cultures, such as those of Korea, Japan, Thailand, and United States (Sung, 1995; Harris et al., 1998; Sharps et al., 1998). The given results suggest that this emic (i.e., culture-specific) Confucian virtue should be viewed as an etic (i.e., universal) construct. The consideration of filial piety as one of the 40 items in the book of The Chinese Culture Connection (1987) and as one of the 57 items in the Schwartz Value Survey (Schwartz, 1992) has, in previous studies, confirmed the universality of this culturally derived specific concept. Although filial practice may be different in many cultures, a universal (etic) nature of this variable has already been established, and this has provided an empirical base for studying it beyond Confucian cultures.

Nowadays, filial piety is one of the most basic universal virtues found in different cultures; it not only determines norms and beliefs within the family, but it also shapes the social and ethical directions for maintaining a stable society (Low and Ang, 2012). In modern psychological studies, filial piety is defined as a cognitive script for social exchanges in intimate relationships which shape individuals’ attitudes. However, the existing research has produced conflicting findings over whether filial piety is beneficial or harmful to individual development (e.g., inhibiting the individual’s independence, suppressing creativity, eliminating personal desires, and interests).

The Dual Filial Piety Model (DFPM; Yeh, 2003) integrates these conflicting findings and is comprised of two higher-order factors that correspond to the two focal filial piety attributes—reciprocal (need of interpersonal relatedness) and authoritarian (need of social belonging and collective identity)—which have been shown to have distinct implications on social adaptation and individuals’ psychological functioning (Yeh and Bedford, 2004; Yeh et al., 2013; Chen, 2014; Chen et al., 2015). The DFPM proposes to try to transform filial piety from its Chinese culture-specific norms to a *contextualized personality* construct that is represented by culturally-sensitive psychological schemas of parent–child interaction.

Such matrix of social relations could be an object for investigation in any culture. Contextualized personality refers to stable patterns of thoughts, feelings, and behaviors that occur within a given context (Bedford and Yeh, 2019). Its authors claim that DFPM represents four possible modes of parent–child interactions: *balanced mode, reciprocal mode, authoritarian mode*, and *non-filial mode*. Interestingly, researchers have considered comparing filial piety and attachment style: *secure attachment style* corresponds to the balanced mode (high RFP and high AFP), the *avoidant attachment style* represents the non-filial mode (low RFP and low AFP), and the *ambivalent attachment style* refers to both reciprocal (high RFP and low AFP) and authoritarian (high AFP and low RFP) modes (see: Bedford and Yeh, 2019).

Two facets of DFPM could be measured by the Dual Filial Piety Scale (DFPS) consisting of 16 items (Yeh and Bedford, 2003). It must be noted that reciprocal and authoritarian factors are not in opposition but coexist in the mind of the person and may cause the same effect (Bedford and Yeh, 2019). The results show that both factors could be analyzed on different levels: as individual motives (reciprocal vs. authoritarian) in the context of parent–child relations, the horizontal vs. vertical structural properties of the parent–child interactions, and the core vs. the changing aspect of social changes in filial norms and differences across societies in the expression of filial piety (Tsao and Yeh, 2019). At the cross-cultural level, RFP (representing psychological prototype) and AFP (representing cultural prototype) describe two fundamental psychological schemas that can be identified as universal etic construct (Yeh et al., 2009).

Reciprocal filial piety (RFP) meets the psychological need for emotional connectedness in social relations and is created by long-term positive interaction with parents in everyday life. The practices of RFP, i.e., respecting, caring for, and attending to one’s parents, fulfill the internal desire for relatedness between two individuals with a horizontal (equal) relationship. The concept of RFP is convergent with the Western ideas of equality and democracy (Yeh et al., 2013). Because RFP is motivated by gratitude and a desire to repay one’s parents for their efforts in the child-raising process (Yeh et al., 2013), its effects are generally positive, producing better interpersonal relationships with parents (Yeh and Bedford, 2004) and higher life satisfaction (Chen, 2014).

The second factor, authoritarian filial piety (AFP), is based on role obligations, compliance with and subordination to parental authority, and is driven by the need for collective identification in vertical (hierarchical) relationships. Individuals with AFP are accustomed to following the rigid social definition of being a son or a daughter consistent with parental demands and expectations (Yeh et al., 2013). Some authors claim that the effects of AFP are generally more negative because they are related to increased levels of depression, anxiety, and aggression (Yeh, 2006). Although the expectation of conformity to parental wishes and the restrictions associated with AFP may block individual autonomy, the willingness to sacrifice for the family may help to maintain harmony within the family and thus benefit the family as a whole system (Yeh and Bedford, 2004; Yeh, 2006).

Filial piety is aimed at deploying notions of social responsibility to create peace and harmony in society, as it underscores the importance of social relationships, solidarity, justice, and sincerity. This applies also to business transactions because a good leader has an obligation to cultivate and improve loyalty and morality. In this case, Yang (1996) views filial piety as a social orientation through which reciprocity functions as a *self-reinforcing power*. Bedford and Yeh (2019) see filial piety as a contextualized personality construct connecting individual-level motivations or goals to their social context.

Thus, family obligation has also been found to correlate with greater academic achievement (Fuligni and Zhang, 2004). A recent study in Hong Kong found that RFP and AFP represent two different motivational beliefs, each with its own influence on academic success (Chen and Wong, 2014). However, results of other different studies are confusing: RFP tends to be positively associated with a higher level of education and positively correlates with life satisfaction (Wong et al., 2010), but AFP seems to be positively associated with less education and lower life satisfaction because of self-suppression (Yeh, 2006). Also, other results showed that RFP was positively associated with academic achievement *via* the satisfaction of the need for autonomy, while the AFP was negatively associated with academic achievement (Zhou et al., 2020). Furthermore, the authors suggest that while filial piety is embedded in Eastern settings, it can be applied to a global context, where RFP in society, but not AFP, is related to student academic achievement (*via* autonomy).

However, there is no study investigating the influence of RFP and AFP on school attitude and functioning in a different cultural context.

## Hypotheses

In our study, we decide to investigate the influence of filial piety (RFP and AFP) as an independent variable on study engagement and study satisfaction (dependent variables) in the more individualistic and egalitarian Polish culture versus the more collectivistic and hierarchical Vietnamese society (culture as moderator).

**FIGURE 1:**
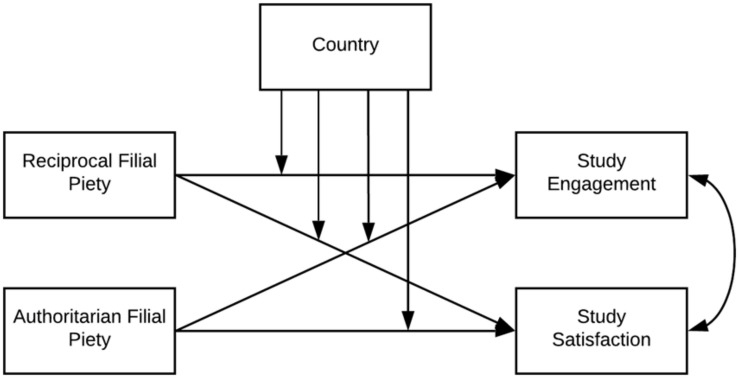
Theoretical model tested in the study.

The aim of the study was to describe the moderating effect of culture on the relationship between filial piety and attitudes toward study. In other words, the main purpose was to investigate the cultural similarities and differences in the psychological functions of RFP and AFP as predictors of study engagement and study satisfaction. The theoretical model tested in the given research is showed in Figure 1.

Regarding the theoretical background and our model, which we tested in the study, we stated four hypotheses in which we looked for the moderating effect of culture (Polish vs. Vietnamese).

H1: The country in which one lives affects the relationship between RFP and study engagement; reciprocal filial piety correlates more strongly with studies engagement in the case of Polish students than it does among Vietnamese students.

H2: The country in which one lives affects the relationship between AFP and study engagement; authoritarian filial piety correlates more strongly with study engagement among Vietnamese students than it does among Polish students.

H3: The country living which one lives affects the relationship between RFP and study satisfaction; reciprocal filial piety correlates more strongly with study satisfaction among Polish students than it does among Vietnamese students.

H4: The country in which one lives affects the relationship between AFP and study satisfaction; authoritarian filial piety correlates more strongly with study satisfaction among Vietnamese students than it does among Polish students.

## Materials and Methods

### Participants and Procedure

The survey was conducted among college students, some of whom received extra course credit points. Participation in the study was voluntary and anonymous. We followed APA standards and the Declaration of Helsinki during the preparation and conduction of the study. The study protocol was approved by the Ethics Board for Research Projects at the Institute of Psychology, University of Gdańsk.

The Polish sample comprised *N* = 310 students of social sciences (75% female, *M*_*age*_ = 20.69, *SD* = 3.10) from the University of Gdańsk (UG). The Vietnamese sample comprised *N* = 297 students (77% female, *M*_*age*_ = 19.23, *SD* = 1.02) from the University of Social Sciences and Humanities (USSH) in Hanoi.

Bilingual psychologists with scientific experience created country-level versions of the scales using the back-translation procedure. The English versions of the scales were used as the basis for all translations. Participants completed a paper and pencil version of the questionnaires (Polish or Vietnamese version, respectively) and answered demographic questions.

### Measures

Participants were asked to complete the Dual Filial Piety Scale (DFPS) developed by Yeh and Bedford (2003). We used the Polish adaptation by Różycka-Tran et al., unpublished and the Vietnamese adaptation by Truong et al. (2020). Both adaptations were authorized by the authors of the original version of the tool. In the study, we also used the Utrecht Work Engagement Scale for Students (UWES–S9) developed by Schaufeli et al., 2002; see also Carmona-Halty et al., 2019) and the Study Satisfaction Scale—a modified version of the Career Satisfaction Scale developed by Greenhaus et al. (1990).

The Dual Filial Piety Scale (DFPS) consists of 16 items. Eight items measure reciprocal (RFP), and eight items measure authoritarian (AFP) filial piety. Respondents were asked to indicate on a scale from 1 to 7 the importance of each statement. Examples of items measuring RFP include the following statement: “Be grateful to parents for raising you”; and AFP includes the following item: “Live with parents even after marriage.” In the current study internal consistency coefficients (Cronbach’s alphas) were strong for both scores in both national samples: 0.87 and 0.85 for RFP subscale in the Polish and Vietnamese samples, respectively; 0.77 and 0.84 for AFP subscale in the Polish and Vietnamese samples, respectively.

The Utrecht Work Engagement Scale for Students (UWES–S9; Schaufeli et al., 2002; Carmona-Halty et al., 2019) is a widely used tool to assess study engagement. The scale consists of nine items grouped into three dimensions with three items each: vigor (e.g., “When I’m doing my work as a student, I feel bursting with energy”), dedication (e.g., “I am proud of my studies”), and absorption (e.g., “I feel happy when I am studying intensely”). All items are scored on a frequency rating scale ranging from 0 (*never*) to 6 (*always*). In the current study, we used only the general score of the scale to indicate overall study engagement. Internal consistency coefficients (Cronbach’s alphas) were strong in both samples: 0.90 and 0.89 in the Polish and Vietnamese samples, respectively.

The Study Satisfaction Scale is the student version of the widely accepted measure of career satisfaction developed by Greenhaus et al., 1990; see also Spurk et al., 2011). It is a five-item self-report scale measuring subjective feelings of study-related success (e.g., “I am satisfied with the progress I have made toward meeting my overall study goals”). All items are scored on a 7–point Likert scale ranging from 1 (*strongly disagree*) to 7 (*strongly agree*). In the current study internal consistency coefficients (Cronbach’s alphas) were strong in both samples: 0.86 and 0.88 in the Polish and Vietnamese samples, respectively.

### Statistical Analysis

First, we needed to determine whether the three scales we used in the study measured the same constructs in both countries, i.e., that they demonstrated measurement invariance across the Polish and Vietnamese samples. Thus, we assessed the three scales’ cross-country equivalence through multigroup confirmatory factor analysis (MGCFA). In the beginning, the factorial structure of each scale was assessed separately for Polish and Vietnamese samples using CFA. To assess the fit of the models, we followed Brown (2015), using the following criteria: CFI > 0.90 and RMSEA < 0.08 (e.g., Brown, 2015). However, Kenny et al. (2015) showed that RMSEA often underestimates fit when the degree of freedom is small, so we used an SRMR criterion <0.08 for the UWES-9S and the Study Satisfaction Scale.

In the steps following, we tested the measurement invariance of the three scales we used in the study in Poland and Vietnam. In cross-country research, we usually estimate three levels of invariance: configural, metric, and scalar. Each of them is defined by the parameters that are constrained to be equal across samples (Milfont and Fisher, 2010; Beaujean, 2014). Configural invariance is present if in all groups the measurement model is built of the same number of factors that consist of the same indicators; metric invariance is determined when factor loadings are equal across the groups; and scalar invariance requires that factor loadings and all intercepts are equal across the groups. It is also possible to determine partial invariance, which is considered to be sufficient for cross-group comparisons (Byrne et al., 1989). Partial invariance requires that the parameters of at least two indicators per construct are equal across the groups.

We started the measurement invariance investigation by testing for configural invariance across the Polish and Vietnamese samples. To identify subsequent levels of measurement invariance (metric and scalar), we used the following cut-off criteria: ΔCFI ≤ 0.01 (see Chen, 2007). The R environment (R Core Team, 2018) supported by the lavaan package (Rosseel, 2012) was used to conduct measurement invariance analysis using maximum likelihood with robust standard errors estimation (MLM).

We next compared the significance of differences between the average results of the studied variables between Polish and Vietnamese students. For this purpose, we used the *t* test for independence samples. We also conducted a linear regression analysis to test the hypothesis about the moderation role of a country in the relationships between filial piety and study attitudes (engagement and satisfaction). Our model included two independent variables (RFP and AFP): one moderator (country) and two dependent variables (study engagement and study satisfaction) (see Figure 1). Finally, to illustrate the moderation effect, we conducted a linear regression analysis separately on the Polish and Vietnamese samples.

## Results

### Measurement Invariance of the Scales Used in the Study

First, we conducted a series of CFAs (separate for each country) testing a two-factor model of the DFPS, a one-factor model of the UWES-9S, and a one-factor model of the Study Satisfaction Scale. As can be seen in Table 1, the CFI as well as RMSEA and SRMR (for models with a small degree of freedom) values suggested a good fit in both countries.

**TABLE 1:** CFA fit statistics for the structural models of the three scales used in the study.

Country	Measure	χ^2^	*df*	CFI	RMSEA	SRMR
Poland	Dual filial piety scale (two-factor model)	249.94	102	0.90	0.068	0.072
	UWES-S9 (one-factor model)	112.79	25	0.93	0.106	0.071
	Study satisfaction scale (one-factor model)	25.61	4	0.97	0.132	0.039
Vietnam	Dual filial piety scale (two-factor model)	203.21	102	0.91	0.060	0.073
	UWES-S9 (one-factor model)	83.99	25	0.93	0.089	0.051
	Study satisfaction scale (one-factor model)	33.92	4	0.96	0.159	0.047

*χ^2^, chi square; *df*, degrees of freedom; CFI, comparative fit index; RMSEA, root mean square error of approximation; SRMR, standardized root mean square residual.*

Next, we conducted a three-level measurement invariance test for each scale. Table 2 presents the global fit coefficients for configural, metric, scalar, and partial scalar equivalent. These results allow us to conclude that all three of the measures we used in the study reached partial scalar invariance across samples, which allows us to make cross-country comparisons.

**TABLE 2:** Global fit measures in measurement invariance tests for the three scales used in the study.

Measure	Level of invariance	χ^2^	*df*	CFI	Δ CFI
Dual filial piety scale	Configural (equal form)	451.07	204	0.91	–
	Metric (equal factor loadings)	480.27	218	0.90	0.01
	Partial scalar (equal intercepts but not all) ^a^	495.88	224	0.89	0.01
	Scalar (equal intercepts)	1228.27	232	0.65	0.25
UWES-S9	Configural (equal form)	195.82	50	0.93	–
	Metric (equal factor loadings)	210.05	58	0.93	0.00
	Partial scalar (equal intercepts but not all) ^b^	215.11	60	0.92	0.01
	Scalar (equal intercepts)	618.93	66	0.78	0.15
Study satisfaction scale	Configural (equal form)	61.56	8	0.96	–
	Metric (equal factor loadings)	83.55	12	0.95	0.01
	Partial scalar (equal intercepts but not all) ^c^	95.50	13	0.94	0.01
	Scalar (equal intercepts)	167.41	16	0.90	0.05

*χ^2^, chi square; *df*, degrees of freedom; CFI, comparative fit index; ^*a*^Intercepts for item 1, 2, 3, 5, 7, 9, 10, and 16 were released; ^*b*^Intercepts for item 1, 2, 6, 7, 8, and 9 were released; ^*c*^Intercepts for item 1, 2, and 4 were released.*

### Polish-Vietnamese Differences in the Examined Variables

As can be seen in Table 3, Vietnamese students scored higher on both reciprocal and authoritarian filial piety subscales compared to Polish students. At the same time, Polish students reported higher study satisfaction compared to their peers in Vietnam. However, there were no significant differences in study engagement between students from both countries.

**TABLE 3:** Differences between the average results of the studied variables between Polish and Vietnamese students.

Variable	Poland		Vietnam		*t*(*df*)	*P*	*Cohen’s d*
	*M*	*SD*	*M*	*SD*			
Reciprocal filial piety	5.76	0.87	6.14	0.71	−5.80 (605)	<0.001	0.49
Authoritarian filial piety	2.81	0.91	3.95	1.03	−14.43 (605)	<0.001	1.17
Study engagement	3.17	1.11	3.16	0.99	0.11 (605)	=0.916	0.01
Study satisfaction	4.45	1.26	3.90	1.27	5.37 (605)	<0.001	0.43

**N*_*Poland*_ = 310, *N*_*Vietnam*_ = 297.*

### Moderating Effect of Country on the Relationship Between Filial Piety and Study Attitudes

**FIGURE 2:**
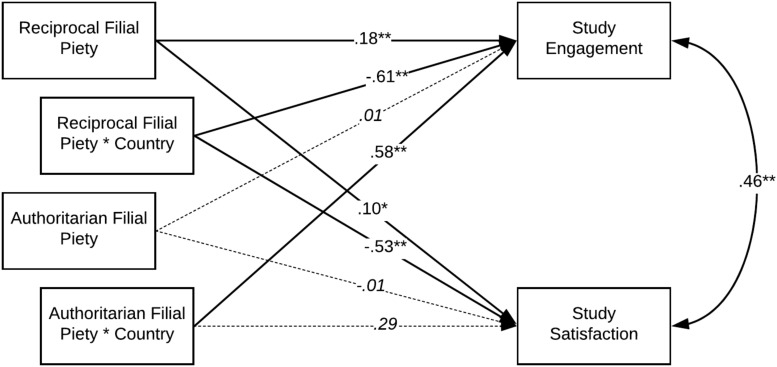
Results of the regression analysis – standardized coefficients.

As can be seen in Figure 2, according to H1 and H2, the country of living has a significant moderating effect on the relationship between reciprocal filial piety and study engagement (β = −0.61, *p* < 0.01) as well as on the relationship between authoritarian filial piety and study engagement (β = 0.58, *p* < 0.01). After adding interaction components to the model, the adjusted *R*^2^ value increased significantly from 0.02 to 0.05 (F Change = 8.12, *p* < 0.01). As expected, RFP has a significant positive relationship with study engagement, but only in the group of Polish students. The opposite result was found for AFP, which is related to study engagement, but only in the group of Vietnamese students (see Table 4).

**TABLE 4:** Results of the regression analysis separately for the Polish and Vietnamese samples.

Dependent variable	Country	Predictors	B	SE	Beta	Model summary
Study engagement	Poland	Reciprocal filial piety	0,25	0,08	0,20**	*F*(2, 309) = 5.33, *p* < 0.01, *R*^2^ = 0.03
		Authoritarian filial piety	−0,07	0,08	−0,06	
	Vietnam	Reciprocal filial piety	0,15	0,08	0,11	*F*(2, 296) = 6.42, *p* = 0.75, *R*^2^ = 0.04
		Authoritarian filial piety	0,13	0,06	0,14*	
Study satisfaction	Poland	Reciprocal filial piety	0,28	0,09	0,20**	*F*(2, 309) = 5.80, *p* < 0.01, *R*^2^ = 0.03
		Authoritarian filial piety	−0,02	0,09	−0,01	
	Vietnam	Reciprocal filial piety	−0,07	0,11	−0,04	*F*(2, 296) = 0.29, *p* = 0.75, *R*^2^ = 0.00
		Authoritarian filial piety	0,05	0,08	0,04	

According to H3, the country of living has a significant moderating effect on the relationship between RFP and study satisfaction (β = −0.53, *p* < 0.01). After adding interaction components to the model, the adjusted *R*^2^ value increased significantly from 0.01 to 0.06 (F Change = 17.49, *p* < 0.01). As expected, RFP has a significant positive relationship with study satisfaction but only in the group of Polish students. However, the results do not support hypothesis H4. In both countries, there is no significant relationship between AFP and study satisfaction (see Table 4).

It is worth noting that study engagement correlates more positively with study satisfaction in the group of Polish students (*r* = 0.56) than among their Vietnamese peers (*r* = 0.38). In addition, it should be noted that Vietnamese students in the current study declared significantly lower satisfaction with academic achievements than did the Polish students. These two results can help find an explanation for the lack of relationship between filial piety and study satisfaction in the group of Vietnamese students.

## Discussion

From the perspective of DFPM, the major developmental task of children, instead of just the personal autonomy whose inculcation is prioritized especially in western cultures, is to form self-volition by integrating numerous social roles as in eastern cultures. Shaping the social matrix is very important because the parent–child relationship becomes the foundation for future social relations with others and helps enrich a more complex identity composed of different social roles (Bedford and Yeh, 2019). It means that filial piety mode established in early childhood influences future relations in school between children and teachers, being an important predictor of academic achievement and study satisfaction.

Our study contributed new results to the theory about the cross-cultural context of teacher–student relations. The cross-cultural comparison studies should not focus on the level of filial piety in every society but should identify cultural similarities and differences in the psychological functions of RFP and AFP (Tsao and Yeh, 2019). Our results show that in more individualistic cultures, the RFP (reciprocal mode) is a stronger predictor of study engagement and study satisfaction; however, the AFP (authoritarian mode) is a better factor to predict study engagement in more collectivistic cultures. So, the implications for practice could concern reciprocity enhanced during the raising of children in a more individualistic culture; however, the authoritarian mode should be enhanced in a more collectivistic society. What is more, only RFP is positively correlated with study satisfaction in an individualistic culture; different predictors of study satisfaction should thus be investigated in more collectivistic cultures in future studies. Our findings revealed that in different cultures, different aspects of filial piety should be underlined by parents in the context of future academic achievements of their children.

The correlation between AFP and study satisfaction in both cultures could result from the definition of AFP: filling social roles and fulfilling social obligations requires self-suppression, where satisfaction is not so important a motivator. In Vietnam, hewing to filial piety norms has nothing in common with personal satisfaction—these are two different motives of psychological functioning and behavior in a collectivistic culture. However, our results show that in an individualistic and egalitarian culture, RFP influences both study engagement and study satisfaction, and it is therefore important to enhance reciprocity in raising individualistic children.

Our findings are consistent with a cross-cultural study by Li et al. (2010) who analyzed Chinese and European American young adults with respect to how they perceive maternal socialization goals (self-development, filial piety, and collectivism), parenting styles (authoritative, authoritarian, and training), and the social-emotional adjustment (self-esteem, academic self-efficacy, and depression). They found cross-cultural similarities between perceived maternal authoritative parenting and socioemotional adjustment (e.g., higher self-esteem and higher academic self-efficacy). However, only Chinese participants declared perceived maternal authoritarian parenting styles as related to socioemotional adjustment (e.g., higher academic self-efficacy). It means that a parenting style shaped by socialization goals is crucial for socio-emotional adjustment (i.e., academic achievement and satisfaction), although it is moderated by culture.

The final conclusion is that the prevention and intervention strategies or techniques intended for children with school problems should be appropriate to the culture and addressed to the parents of children in kindergarten and later to very early-stage education teachers. The intervention should be through a combination of both in-school and external factors. Teachers should promote high expectations and strong teacher–student relationships in a collectivistic society because high expectations increase students’ sense of self-efficacy and motivation, which improves achievement and aspirations. In more individualistic cultures, achievements could be improved by quality teacher–student relationships based on support and the emotional sensitivity of the teacher. The results of studies based on the DFPM may stimulate practical applications and policy development in the domain of success and failure in the academic environment.

## Limitations and Future Studies

The main limitation of the study is student samples that are not representative of the whole of the respective countries. Further studies should use a sample with a broader range of ages and occupations; for example, employees in different organizations. Currently, there is a growing interest in human capital analytics, which consists of explaining and predicting the efficiency of the organization by means of employee data. One of the important sources of information on the functioning of the organization and people employed in it are employee opinion surveys. It would be interesting to investigate the role of filial piety in perceived working conditions and work engagement through employees’ attitudes toward the organization.

In future studies, the construct validity of the DFPS-V should be tested by examining the relationship of filial piety with other validated measurements using other assessment methods such as children’s ratings of their parents and as ratings done by parents.

Future studies examining filial piety ratings by both parents and children would be a significant contribution to the knowledge about this important social and psychological concept on both the individual and cultural level.

## Data Availability Statement

The datasets generated for this study are available on request to the corresponding author.

## Ethics Statement

The studies involving human participants were reviewed and approved by Ethics Board for Research Projects at the Institute of Psychology, University of Gdańsk. Written informed consent for participation was not required for this study in accordance with the national legislation and the institutional requirements. Written informed consent was obtained from the individual(s) for the publication of any potentially identifiable images or data included in this article.

## Author Contributions

JR-T and PJ designed the study, gathered data, wrote the manuscript, searched references, and organized fundings for the manuscript. PJ and MO analyzed and interpret the data. TKHT gathered data and gave comments. All authors contributed to the article and approved the submitted version.

## Conflict of Interest

The authors declare that the research was conducted in the absence of any commercial or financial relationships that could be construed as a potential conflict of interest.
